# ^18^F-FDG PET/CT Radiomics for Preoperative Prediction of Lymph Node Metastases and Nodal Staging in Gastric Cancer

**DOI:** 10.3389/fonc.2021.723345

**Published:** 2021-09-13

**Authors:** Qiufang Liu, Jiaru Li, Bowen Xin, Yuyun Sun, Dagan Feng, Michael J. Fulham, Xiuying Wang, Shaoli Song

**Affiliations:** ^1^Department of Nuclear Medicine, Fudan University Shanghai Cancer Center, Fudan University, Shanghai, China; ^2^Department of Oncology, Shanghai Medical College, Fudan University, Shanghai, China; ^3^School of Computer Science, The University of Sydney, Sydney, NSW, Australia; ^4^Department of Molecular Imaging, Royal Prince Alfred Hospital, The University of Sydney, Sydney, NSW, Australia

**Keywords:** lymph nodes metastases, PET/CT, radiomics, N stage, gastric cancer

## Abstract

**Objectives:**

The accurate assessment of lymph node metastases (LNMs) and the preoperative nodal (N) stage are critical for the precise treatment of patients with gastric cancer (GC). The diagnostic performance, however, of current imaging procedures used for this assessment is sub-optimal. Our aim was to investigate the value of preoperative ^18^F-FDG PET/CT radiomic features to predict LNMs and the N stage.

**Methods:**

We retrospectively collected clinical and ^18^F-FDG PET/CT imaging data of 185 patients with GC who underwent total or partial radical gastrectomy. Patients were allocated to training and validation sets using the stratified method at a fixed ratio (8:2). There were 2,100 radiomic features extracted from the ^18^F-FDG PET/CT scans. After selecting radiomic features by the random forest, relevancy-based, and sequential forward selection methods, the BalancedBagging ensemble classifier was established for the preoperative prediction of LNMs, and the OneVsRest classifier for the N stage. The performance of the models was primarily evaluated by the AUC and accuracy, and validated by the independent validation methods. Analysis of the feature importance and the correlation were also conducted. We also compared the predictive performance of our radiomic models to that with the contrast-enhanced CT (CECT) and ^18^F-FDG PET/CT.

**Results:**

There were 185 patients—127 men, 58 women, with the median age of 62, and an age range of 22–86 years. One CT feature and one PET feature were selected to predict LNMs and achieved the best performance (AUC: 82.2%, accuracy: 85.2%). This radiomic model also detected some LNMs that were missed in CECT (19.6%) and ^18^F-FDG PET/CT (35.7%). For predicting the N stage, four CT features and one PET feature were selected (AUC: 73.7%, accuracy: 62.3%). Of note, a proportion of patients in the validation set whose LNMs were incorrectly staged by CECT (57.4%) and ^18^F-FDG PET/CT (55%) were diagnosed correctly by our radiomic model.

**Conclusion:**

We developed and validated two machine learning models based on the preoperative ^18^F-FDG PET/CT images that have a predictive value for LNMs and the N stage in GC. These predictive models show a promise to offer a potentially useful adjunct to current staging approaches for patients with GC.

## 1 Introduction

Gastric cancer (GC) is the fifth most common malignancy and the third leading cause of cancer death worldwide (1, 2). Clinical staging, based mainly on imaging, is critical in determining the best treatment. Involvement of regional lymph nodes with metastases (LNMs) is classified as N0 (no LNM), N1 (1–2 LNMs), N2 (3–6 LNMs), N3a (7–15 LNMs), and N3b (≥ 16 LNMs). The different nodal (N) stage then determines the treatment strategy. Various investigators showed that patients with LNMs have a poor prognosis and a high recurrence rate (3–5). According to the Japanese Gastric Cancer Treatment Guidelines (6), radical gastrectomy with level-2 extended lymphadenectomy (D2 resections) is the standard treatment for GC without LNMs. For patients with advanced stages who cannot undertake surgery, preoperative evaluation of LNMs could provide useful information for determining the appropriate adjuvant therapy, while for patients who are suitable for surgery, accurate detection of LNMs prior to surgery could help in determining the surgical approach and lymph node dissection range. Therefore, the accurate detection of LNMs prior to surgery is required for an appropriate decision-making in GC.

Currently, contrast-enhanced CT (CECT) is used for N staging. Kim et al. (7) reported that the accuracy of CT was 50%–70% for LNMs. Unlike CECT imaging, ^18^F-fluorodeoxyglucose positron emission tomography-CT (^18^F-FDG PET/CT) reflects the glucose metabolism in tumors and can detect disease in lymph nodes that are not enlarged, and may have a higher specificity (8). The PET/CT parameters, however, that include the maximum standardized uptake (SUVmax), metabolic volume (MTV), and total lesion glycolysis (TLG), are affected by the different uptake times (time from isotope injection to PET data acquisition), instrumentation differences (different scanners), and attenuation correction methods. Furthermore, the predictive performance of SUVmax has varied across different researchers (9, 10). Yun et al, albeit with a PET-only scanner, stated that the accuracy of ^18^F-FDG PET/CT in identifying LNMs was unsatisfactory (for N1 metastases: PET: 56%, CT: 69%; for N2 metastases: PET: 72%, CT: 69%; for N3 metastases: both PET and CT: 95%) (11). Now, with the advent of new radiomics methods, we suggest that nodal staging in GC should be re-considered.

Radiomics is an imaging analysis method that maximizes the information obtaining from routine diagnostic images and may detect data that is not readily apparent from the images alone (12). Recent advances in radiomics have provided insights into the accurate prediction of the pre-operative clinical stage. Several studies have shown that a CT radiomics nomogram can predict the N staging in a variety of cancers (13–15). Feng et al. developed a computational clinical decision support system based on CT radiomics to predict the involved LNs in gastric cancer, yielding an accuracy of 71.3% (16). Jiang et al. (17) concluded that the radiomic signature was a powerful predictor of LNMs based on the significant association between the CT radiomic signature and the pathological LN stage in GC. When compared to CT, ^18^F-FDG PET/CT offers an additional advantage of providing metabolic information. Recently, PET/CT radiomics studies have been published on predicting the treatment response, prognosis, and the pathology sub-types (18–20). The predictive value of ^18^F-FDG PET/CT radiomics in the N staging of GC, to our knowledge, has not been widely investigated. In this study, our aim was to develop and validate predictive machine learning models based on ^18^F-FDG PET/CT radiomics to predict the LNMs and specific N stage in GC.

## 2 Materials and Methods

### 2.1 Patients Inclusion Criteria

This study was approved by the Ethics Committee of the Fudan University Shanghai Cancer Center (No. 1909207-14-1910), and the need for the written informed consent was waived. There were 185 patients diagnosed with GC who underwent a total or partial radical gastrectomy at Fudan University Shanghai Cancer Hospital, including 156 GC patients obtained from January 2019 to May 2020 and 29 GC patients recruited from May 2020 to June 2021. These patients were reviewed retrospectively. The TNM staging was conducted according to the American Joint Committee on Cancer TNM Staging Manual, Eighth Edition (21). The inclusion criteria were as follows: (1) patients diagnosed as GC on surgically resected specimens; (2) patients with available clinical features such as sex, age, and tumor size; (3) patients with available ^18^F-FDG PET/CT scan data before surgery; and (4) patients who did not receive neoadjuvant therapy before surgery.

### 2.2 Imaging Protocols and Image Analysis

A total of 161 out of 185 GC patients received dynamic contrast scans with a multidetector spiral CT (Sensation 64; Siemens Medical Systems, Germany). Contrast images were acquired in the arterial (delay time: 30–35 seconds) and portal phases (delay time: 65–70 seconds) after an intravenous injection of 90 ml of iohexol (Omnipaque 300; Amersham, Shanghai, China) at a rate of 3 ml/second. Images were obtained at 120 kV and 200 mA with a 1-mm slice thickness. CT findings of the tumor location, size, perigastric lymph nodes, degree and pattern of enhancement, and distant metastases were analyzed. The size of the tumor was determined according to the maximum diameter of the tumor on the axial/coronal/sagittal images in the contrast phase. Contrast enhancement was graded as mild (< 10 HU), moderate (10–40 HU), and marked (> 40 HU). A perigastric lymph node was considered positive if the shortest diameter was greater than 10 mm or if there was a marked enhancement. The TNM stage of each patient was recorded by two experienced radiologists, and the results were verified by a third radiologist.

^18^F-FDG PET/CT scans were performed using two whole-body PET/CT scanners (Siemens Medical Systems, Biograph 16 mCT Flow, and Biograph 16 mCT) in the Department of Nuclear Medicine. Patients fasted for at least 6 h, and the blood glucose levels were <140 mg/dl. With the Biograph 16 mCT Flow Scanner, scans were acquired 1 h after an intravenous injection of ^18^F-FDG (3.7 MBq/kg). Images were acquired from the skull base to the upper thighs. A low-dose CT scan (120 kV, 140 mA, 5-mm slice thickness) was performed first to provide attenuation correction and anatomical information. Then, PET scan data were obtained and reconstructed with a time-of-flight ordered subset expectation maximization algorithm (iterations 4; subsets 8; image size 168) (22). With the Biograph 16 mCT Scanner, the scan was acquired approximately 1 h after the intravenous administration of 5.18 MBq/kg of ^18^F-FDG. The CT scans were conducted first (120 kVp, 150 mAs, 0.33 s per rotation, thickness of 3.0 mm) and reconstructed to a 512 × 512 matrix “(voxel size: 0.98 × 0.98 × 3.0 mm^3^). Then, PET scans were performed with the parameters (2 min per bed, 2 iterations, 24 subsets, and 2 mm full width at half maximum) without filtering and smoothing to reconstruct the PET images. Two experienced nuclear medicine physicians evaluated the PET/CT images and measured the maximum standardized uptake value (SUVmax) of the primary tumor and any metastases.

### 2.3 PET/CT Radiomics Analysis With Machine Learning

**Figure 1:**
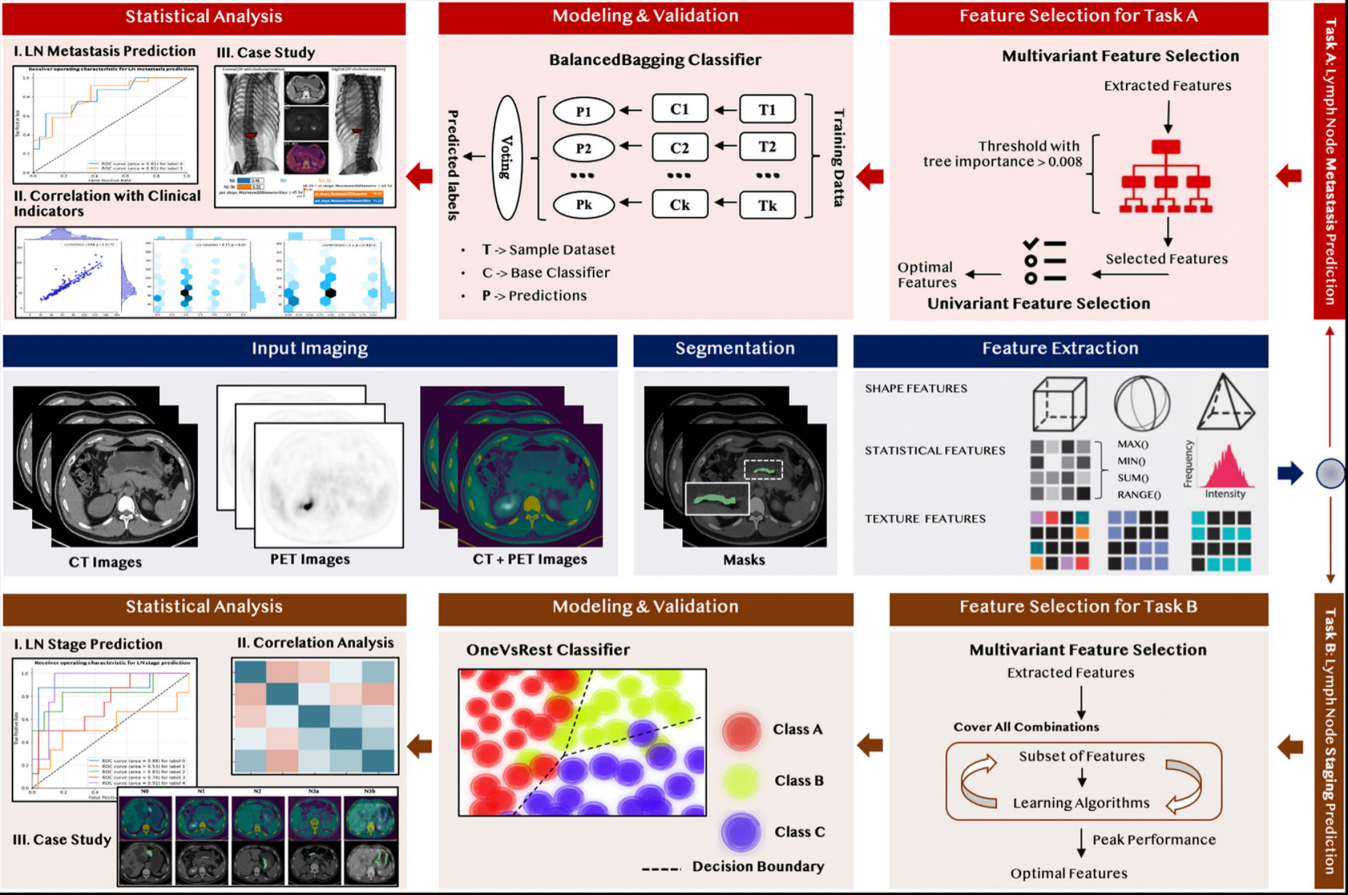
Radiomic flowchart for the prediction of LNMs (task A) and the N stage (task B).

The radiomics analysis workflow is shown in **Figure 1**. There were five principal modules: input image segmentation, radiomic feature extraction, representative feature selection, predictive model construction, and statistical analysis. Firstly, we applied the same input image segmentation and radiomic feature extraction procedure for two different classification tasks, including Task A, predicting the LNMs, and Task B, predicting the N stage. Due to the different nature of the problems, we derived two branches for these two different classification tasks for the remaining principal modules, including feature selection, predictive model construction, and statistical analysis.

### 2.4 Medical Image Segmentation

The volume of interests (VOIs) in the tumor were delineated slice-by-slice with the ITK-SNAP software (version 3.6.0) (23) by the two senior nuclear medicine physicians. If a disagreement occurred, it was resolved by another experienced nuclear medicine physician. As the PET images and CT images were co-registered, only the VOIs of the PET images were individually segmented.

### 2.5 Radiomic Feature Extraction

There were 1,050 PET and 1,050 CT high-quantitative imaging features extracted from the corresponding VOIs. The 1,050 radiomic features included: (1) 18 first-order statistical features that were used to describe the distribution of individual voxel values within the image region defined by the mask through the commonly used and basic metrics without considering the spatial relationships (24); (2) 14 shape features used to describe the geometry properties and the shape of the region of interest (ROI) (25); (3) 56 texture features were extracted to measure the spatial arrangement of the voxel intensities and the intra-lesion heterogeneity, which could be derived from the grey-level co-occurrence matrix (GLCM) and grey-level size zone matrix (GLSZM) (25); and (4) 370 Laplacian of gaussian (LoG) filtered features and 592 wavelets filtered features; both were part of the higher-order statistical features obtained by applying the Laplacian of Gaussian (LoG) transformation and wavelets transformation, individually. Since the higher-order statistics features can suppress the noise and highlight the details in the original images, they are able to extract areas with increasingly coarse texture patterns in a more flexible way. The radiomic feature extraction process was implemented through the PyRadiomics package (24), an open-source package compliant with the Imaging biomarker standardization initiative (26).

### 2.6 Representative Feature Selection

We fused the 2,100 extracted radiomic features with 13 clinical features to form a feature pool before implementing the feature selection module. The feature selection strategy varied for different classification tasks, but both were mainly designed based on the output-driven model, with the aim of capturing the embedded patterns that were beneficial for each classification task.

**Figure 2:**
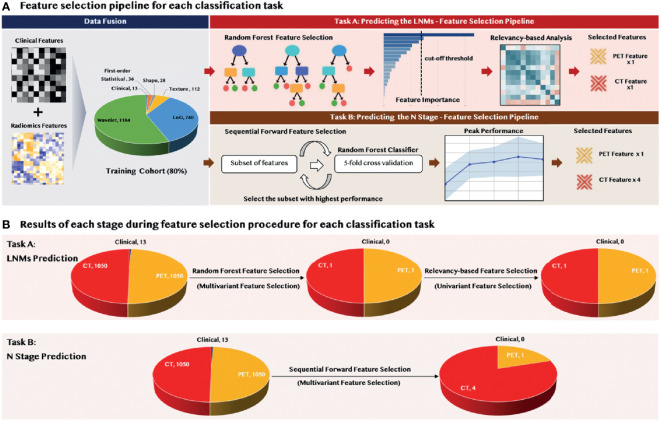
Methodology and the results of feature selection: **(A)** feature selection pipeline, and **(B)** number of selected features during the selection procedure.

As shown in **Figure 2A**, we applied a sequential combination of multivariant and univariant feature selection for predicting LNMs. In the multivariant feature selection, random forest feature selection (with tree importance > 0.008) was used due to its competitive predictive performance, low over-fitting, and easy interpretability. This interpretability was derived by computing the importance of each feature that contributed to the final decision. Then, univariant feature selection was deployed to select the final discriminative features through conducting the relevancy-based analysis using the Pearson correlation method among the selected features and the predicted class.

In predicting the N stage, we applied the sequential forward feature selection method in the multivariant feature selection. Since this algorithm comprehensively covered the combinations of the subsets and automatically selected a subset of the features that offered the best performance on the training dataset, the univariant feature selection was not further required. The performance for each feature subset was evaluated by a 5-fold cross-validation to reduce the risk of overfitting, and the feature set that achieved the peak model performance was considered the final feature set.

### 2.7 Modeling and Validation

Patients, recruited from the 2019–2020 period (n = 156), were allocated into training and validation datasets using stratified methods at a fixed ratio to preserve the proportion of the targets in the original dataset; 80% of the 2019–2020 period patients were assigned to the training set, and the remaining 20% were assigned to the validation set. The 29 additional patients from 2020–2021 were further used to enlarge the independent validation cohorts.

For the prediction of LNMs, a BalancedBagging ensemble classifier (27) incorporated with Adaboost as the base classifier was constructed since it could improve the variance by voting the outcome from multiple base classifiers on variants of the training set and prevent overfitting. For the N stage, the OneVsRest classifier (27) was applied due to its high interpretability and the possibility of gaining knowledge about each class by inspecting its corresponding classes. Furthermore, we used 5-fold cross-validation methods on the training dataset and independent validation methods on the validation set to evaluate the performance and the robustness of each machine learning model. The performance of each model was primarily evaluated by the accuracy and the area under the curve (AUC); the confusion matrix also generated the sensitivity, specificity, positive predicted value (PPV), and negative predicted value (NPV) to detect the existence of bias within the model.

### 2.8 Statistical Analysis

Statistical analysis included a result interpretation of the machine learning model and correlation analysis of the selected radiomic features with pathological features. The Local Interpretable Model-Agnostic Explanations model (LIME) (28) was applied to explain the contribution of each selected feature through its derived weight coefficients to gain insights into the selected features and the predictive model. The LIME model estimated the weight coefficients by observing the changes in the results after eliminating several interpretable components. The changes were measured by the distance from the range center of the resulting changes in the prediction. The closer to the range center, the higher the weight coefficients would be assigned, indicating a better contribution to the final prediction.

We applied the Pearson correlation method that measured the strength and the direction of association between two continuous variables, to evaluate the correlation between the selected radiomic features and the pathological features. The Point-Biserial correlation method was used for the measurement between one continuous variable and one categorical variable. All statistical analyses were performed using the scikit-learn (sklearn) package (27) in Python version 3.6.4, and a two-sided P-value < 0.05 was considered statistically significant.

## 3 Results

### 3.1 Demographics of Patients

The demographic information of 185 patients is summarized in **Table 1**. The included patients underwent open total gastrectomy (n = 103), distal gastrectomy (n = 79), and proximal gastrectomy (n = 3), with D2 lymphadenectomy in accordance with the Japanese guidelines [6], which included lymph node dissection (n > 15) of the perigastric and part of the suprapancreatic area. According to the pathological N stage (pN) of the TNM staging, LNM was divided into five categories: N0: no lymph node metastasis; N1: 1–2 lymph node metastases; N2: 3–6 lymph node metastases; N3a: 7-15 lymph node metastases; N3b: ≥ 16 lymph node metastases. The pathology in 77.8% of the patients was adenocarcinoma and mixed adenocarcinoma in the remainder. There were 136 patients with LNMs, and 49 patients without LNMs. There were 49 patients (26.4%) with N0 stage, 31 patients with N1 (16.8%), 31 patients (16.8%) with N2, 52 patients (28.1%) with N3a, and 22 patients (11.9%) with N3b stage. For ^18^F-FDG PET/CT, the sensitivity was 68.7% and the specificity was 70%, while for CECT the sensitivity was 57.7% and the specificity was 66.7% (see **Table 2**). We maintained the same ratio between different predicted classes for the training set and the validation set as that in the original dataset, and there was no significant difference between the training set and validation set based on a two-sample t-test (p > 0.05).

**Table 1:** Demographic and clinical characteristics of the enrolled patients.

Characteristics		Total Population	N0	N1	N2	N3a	N3b
		(n = 185)	(n = 49)	(n = 31)	(n = 31)	(n = 52)	(n = 22)
Age, median (range)		62 (22–86)	61 (28–81)	63 (36–80)	62 (24–73)	62 (26–86)	66 (22–79)
Gender, n(%)		185	49	31	31	52	22
	Male	127 (68.6)	40 (81.6)	22 (71.0)	20 (64.5)	32 (61.5)	13 (59.1)
	Female	58 (31.4)	9 (18.4)	9 (29.0)	11 (35.5)	20 (38.5)	9 (40.9)
Histopathological Type, n (%)							
	adenocarcinoma	144 (77.8)	42 (85.7)	26 (83.9)	25 (80.6)	41 (78.8)	10 (45.5)
	mixed adenocarcinoma	41 (22.2)	7 (14.3)	5 (16.1)	6 (19.4)	11 (21.2)	12 (54.5)
Lauren Type, n (%)							
	intestinal type	64 (34.6)	23 (46.9)	14 (45.2)	12 (38.7)	14 (26.9)	1 (4.5)
	diffuse type	51 (27.6)	14 (28.6)	5 (16.1)	9 (29.0)	13 (25)	10 (45.5)
	mixed type	70 (37.8)	12 (24.5)	12 (38.7)	10 (32.3)	25 (48.1)	11 (50.0)
Differentiation, n (%)							
	low	85 (45.9)	18 (36.7)	10 (32.3)	12 (38.7)	28 (53.8)	17 (77.3)
	middle-low	58 (31.4)	11 (22.4)	10 (32.3)	13 (41.9)	19 (36.5)	5 (22.7)
	middle	36 (19.5)	16 (32.7)	11 (35.4)	5 (16.1)	4 (7.7)	0 (0.0)
	high	6 (3.2)	4 (8.2)	0 (0.0)	1 (3.3)	1 (2.0)	0 (0.0)
Vascular Tumor Thrombus, n (%)							
	not contain	34 (18.4)	25 (51)	8 (25.8)	1 (3.3)	0 (0.0)	0 (0.0)
	contain	126 (68.1)	12 (24.5)	18 (58.1)	25 (80.6)	51 (98.1)	20 (91.0)
	uncertain	22 (11.9)	12 (24.5)	4 (12.9)	5 (16.1)	0 (0.0)	1 (4.5)
	multiple tumors	3 (1.6)	0 (0.0)	1 (3.2)	0 (0.0)	1 (1.9)	1 (4.5)
Infiltration depth, n (%)							
	lamina propria or submucosa	31 (16.8)	20 (40.8)	5 (16.1)	3 (9.7)	2 (3.8)	1 (4.5)
	muscularis propria	23 (12.4)	9 (18.4)	6 (19.4)	3 (9.7)	4 (7.7)	1 (4.5)
	subserosa	54 (29.2)	10 (20.4)	9 (29.0)	13 (41.9)	17 (32.7)	5 (22.8)
	serosal layer	46 (24.8)	7 (14.3)	4 (12.9)	8 (25.8)	21 (40.4)	6 (27.3)
	fat tissue outside the serosal layer etc.	31 (16.8)	3 (6.1)	7 (22.6)	4 (12.9)	8 (15.4)	9 (40.9)
Nerve invasion, n (%)							
	+	104 (56.2)	16 (32.7)	17 (54.8)	19 (61.3)	37 (71.1)	15 (68.2)
	–	61 (33.0)	30 (61.2)	14 (45.2)	7 (22.6)	7 (13.5)	3 (13.6)
	uncertain	20 (10.8)	3 (6.1)	0 (0.0)	5 (16.1)	8 (15.4)	4 (18.2)
SUVmax_tumor, mean (std)		7.76 (5.93)	5.55 (4.41)	8.66 (5.61)	8.34 (5.02)	9.51 (7.57)	6.48 (3.87)
SUVmax_LN, mean (std)		2.92 (3.72)	1.56 (2.28)	3.32 (3.73)	3.07 (3.17)	3.58 (4.96)	3.60 (2.62)
maximum diameter, mean (std)		4.81 (2.97)	3.76 (2.48)	4.14 (2.43)	4.82 (2.10)	4.95 (2.85)	7.77 (3.89)

**Table 2:** Results for predicting lymph node metastases in independent validation cohorts.

Evaluation	Accuracy	AUC	Sensitivity	Specificity	PPV	NPV
CECT	0.602	–	0.577	0.667	**0.817**	0.380
^18^F-FDG PET/CT	0.692	–	0.687	0.70	0.790	0.576
PET feature	0.770	0.724	0.563	0.844	0.563	0.844
CT feature	**0.852**	0.803	**0.769**	0.875	0.625	**0.933**
**CT + PET**	**0.852**	**0.822**	0.733	**0.891**	0.688	0.911

The bold feature value represented the combined radiomic features that achieved high prediction accuracy for both target classes, while the bold numerical value represented the highest value of each column.

### 3.2 Results of Feature Selection

As shown in **Figure 2B**, feature selection was applied to the 2,100 radiomic features extracted from PET and CT, and the 13 clinical features. Only two radiomic features—CT the Maximum3Ddiameter and PET the Maximum2DdiameterSlice—were selected during the multivariant feature selection for the prediction of LNMs. These two features remained through the relevancy-based feature selection and formed the final discriminative feature set used for the model construction. There were five radiomic features selected through the sequential forward feature selection method for the prediction of the N stage; these included four CT features (one shape; one LoG; two wavelet) and one for PET (wavelet).

### 3.3 Performance of Radiomic Features

**Figure 3:**
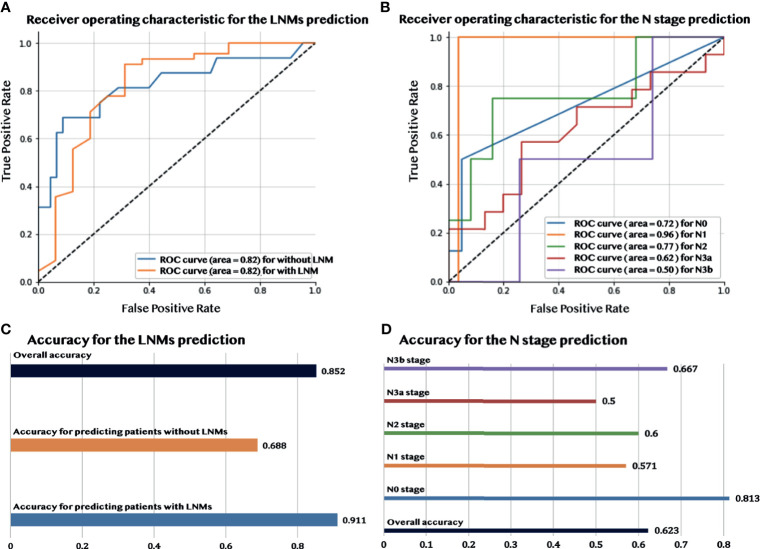
The performance of predicting LNMs and the N stage. **(A)** The AUC curve for predicting LNMs. **(B)** The AUC curve for predicting the N stage. **(C)** Accuracy of the prediction of LNMs. **(D)** Accuracy of the prediction of the N stage.

**Figures 3A, C** show that during the validation process, the model had a good performance in predicting LNMs with an overall accuracy of 85.2% and AUC of 82.2%. More detailed information about the model performance, including sensitivity (73.3%) and specificity (89.1%), are shown in **Table 2**. Furthermore, the predictive model detected an additional 19.6% LNMs missed with CECT in the validation group, and 35.7% with ^18^F-FDG PET/CT. The same evaluation procedure was applied for the model used to predict the N stage. The overall model accuracy was 62.3%, and the AUC was 73.7% (see **Figure 3B**). The model showed a competitive discrimination of the N stage (N0:72%, N1:96%, N2:77%, N3a:62%, and N3b:50%), and the detailed accuracy for each stage is outlined in **Figure 3D**. The overall accuracy for N stage prediction with CECT was 18.2%, and it was 35% for ^18^F-FDG PET/CT in the validation set. In the validation group of N stage prediction, there were 57.4% that were incorrectly staged with CECT and 55% that were incorrect with ^18^F-FDG PET/CT, but which had the correct N stage with the radiomic model.

### 3.4 Feature Analysis and Interpretation

**Figure 4:**
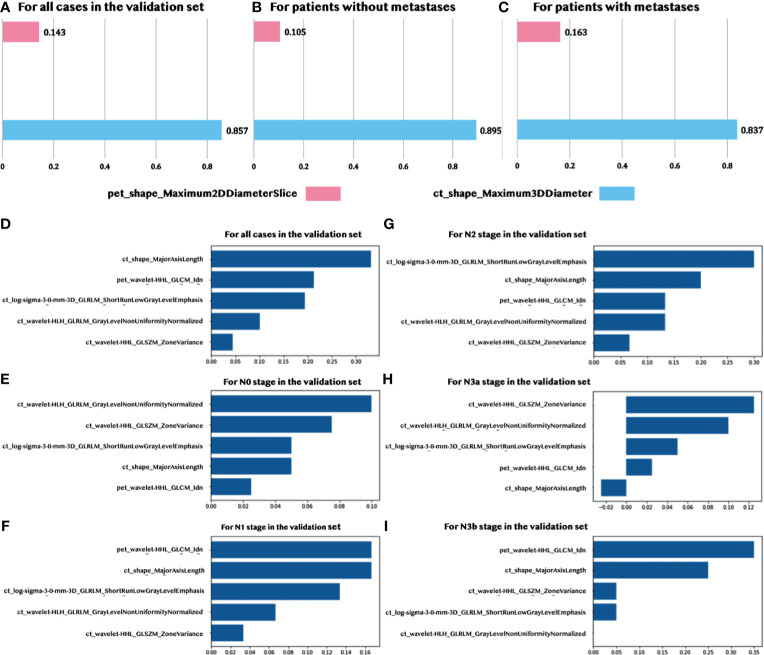
Normalized feature importance. **(A–C)** Feature importance in predicting LNMs for all validation patients and patients with/without metastases. **(D–I)** Feature importance in predicting the N stage for all validation patients and patients with five N stages (N0, N1, N2, N3a, and N3b).

There were two features, one CT feature (ct_shape_Maximum3DDiameter) and one PET feature (pet_shape_Maximum2DDiameterSlice), which were identified by the sequentially combined multivariant and univariant feature selection process for predicting LNMs. The Maximum3DDiameter feature was used to define the largest pairwise Euclidean distance between the tumor surface mesh vertices. The Maximum2DDiameterSlice feature was a similar feature; however, it only defined the distance in the row-column (generally axis) plane. The statistics of these two selected quantitative features are summarized in **Supplementary Table S1**. The contribution of each selected feature in the prediction of LNMs is shown in **Figures 4A, B**, and **C** through the normalized importance calculated by the LIME model (28) in three different situations, including for: (1) all the patients in the validation set, (2) patients without metastases, and (3) patients with metastases. The CT feature had a higher contribution, when compared to the PET feature, in predicting LNMs in these three situations with the normalized importance of 86%, 90%, and 84%, sequentially.

We identified five features in the prediction of the N stage. These included four CT features and one PET wavelet feature. The detailed explanation of these features, including the definition and the calculated formula, are summarized in **Supplementary Table S2**. According to **Figure 4D**, the CT shape feature dominated the contribution to predicting the N stage in the validation set. The contribution of the CT shape feature was very similar to the only PET feature (see **Figures 4E–I**). Both features contributed more to predicting the N1 stage, N2 stage, and N3b stage with a lesser contribution to N0 and N3a.

### 3.5 Case Studies

**Figure 5:**
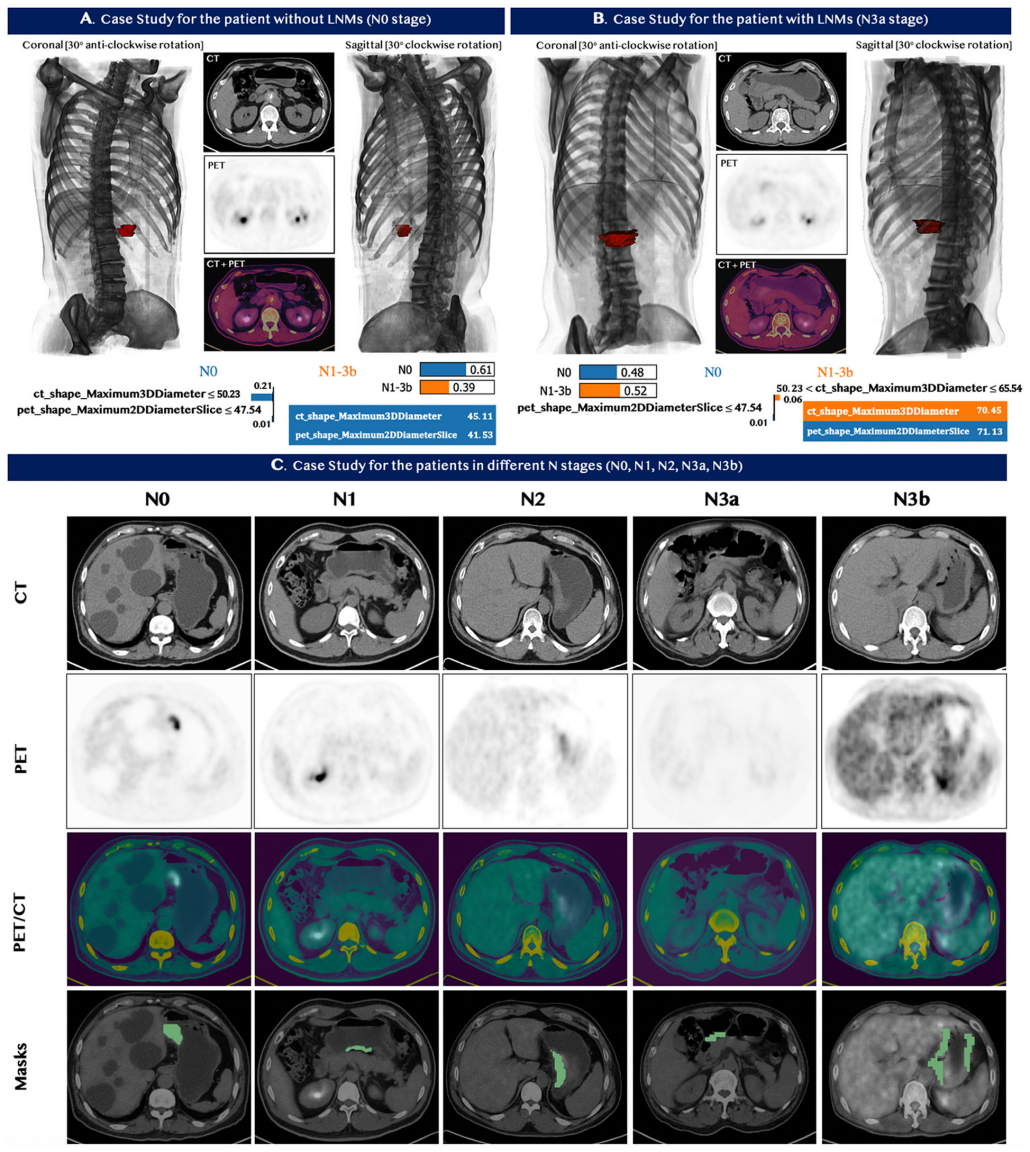
Case studies for seven patients with GC. Top Panels: **(A)** patient with no lymph nodes metastases. **(B)** patient with lymph nodes metastases. The image at the bottom of **(A, B)** contains the feature value of the patients and the corresponding LIME interpretation. The top left and top right sections in panel **(A, B)** demonstrated the 3D model constructed based on the input CT and PET images from different viewpoints, while the red section represented the tumor of the patients. Our predictive model correctly identified the status for both patients in panel **(A, B)**. **(C)** Bottom Panel - Five patients with different stages N0, N1, N2, N3a, and N3b from left to right. Our machine learning model predicted the N stage of the five patients accurately. ^18^F-FDG PET/CT, however, did not detect LNMs in all five patients; and CECT also did not assess the N stages correctly.

Two typical cases were chosen by the domain experts—one patient with and one without metastases—to illustrate the performance of our model in predicting LNMs. The detailed medical information, including the CT and PET images and 3D models for each patient, are shown in **Figures 5A, B**. The value of the selected features for each patient is indicated in the table at the bottom of panels A and B. The contribution of each feature is explicitly revealed by the LIME model through the weight coefficients listed in the bar chart of each panel. The model quantitatively combined the selected features with their diverse weight coefficients for the final prediction and correctly predicted both cases. We also chose five cases to showcase the model performance for the prediction of the N stage. The PET/CT images and the segmentation section are shown in **Figure 5C**. In all five patients, our machine learning model predicted the N stage accurately. In comparison, ^18^F-FDG PET/CT did not detect LNMs in all five patients, and CECT also did not stage the N stages correctly.

### 3.6 Correlation With Pathological Features

**Figure 6:**
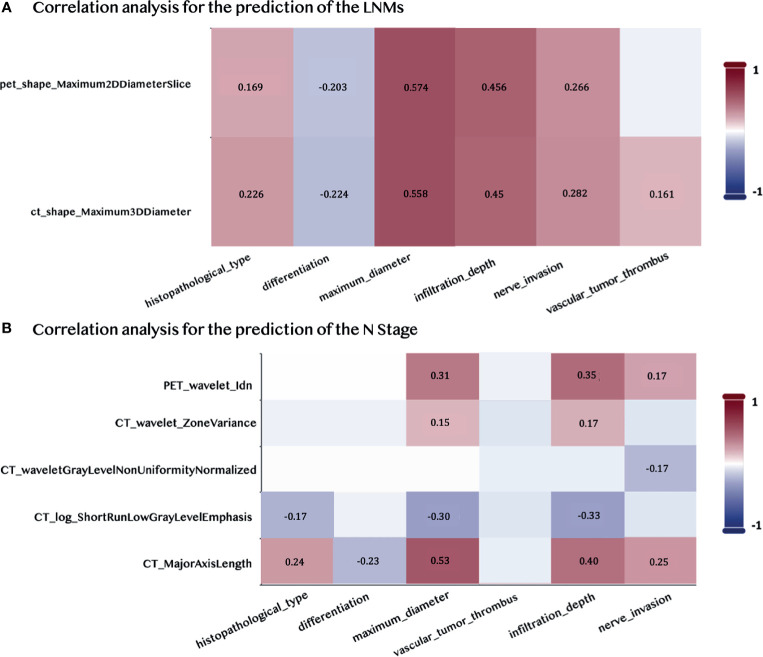
Pearson Correlations between the selected PET/CT features and the pathological features. **(A)** Correlation analysis for predicting LNMs. **(B)** Correlation analysis for predicting the N stage. Pairwise correlations with p < 0.05 are shown in the figure.

We computed the Pearson correlation between the selected radiomic features and the pathological features that were commonly used for the diagnosis of the LNMs to underline the reliability and the significance of two selected features in the prediction of the LNMs. The CT feature was significantly correlated to the vascular tumor thrombus, nerve invasion, histopathological type, differentiation, and infiltration depth (p < 0.05), which explained its high contribution to the final prediction, as shown in **Figure 6A**. The Pearson correlation between the five selected radiomic and pathological features used to predict the N stage is shown in **Figure 6B**. It showed that the PET/CT radiomic features were also significantly correlated (p < 0.05) to the pathological features such as infiltration depth. The detailed P-value for the correlation analysis were summarized in **Supplementary Figure S1**.

## 4 Discussion

Our main findings are as follows: (1) We developed and validated (AUC 82.2%) a binary predictive model using two ^18^F-FDG PET/CT radiomic features to predict LNMs preoperatively. This model might allow clinicians to identify patients with a high risk of LNMs and thus assist diagnosis and decision-making. (2) We developed and validated (AUC 73.7%) a radiomics multiclass predictive model using ^18^F-FDG PET/CT to identify the N stage prior to surgery.

The reported sensitivity (57.7%) and specificity (66.7%) of CECT in our study were similar to a previous work (7). On CECT, enlarged lymphs were not always metastatic, and small lymph nodes could be metastatic, hence, the predictive performance of CECT in detecting LNMs is sub-optimal. Since ^18^F-FDG PET/CT can detect disease in lymph nodes that are not enlarged, more recent clinical guidelines suggest that it might improve GC staging (29). Previous studies showed that a high ^18^F-FDG uptake could be associated with LNMs (30–32). The thresholds of SUVmax, however, varied significantly across different studies. In our study, the performance of ^18^F-FDG PET/CT in predicting LNMs was relatively inferior, especially with a low negative predicted value (NPV) of 57.6%. Our results were consistent with the previous study (33). The reasons might be that: (1) the resolution of ^18^F-FDG PET was limited, which might miss some positive uptake of small LNs; (2) some LNMs presented no ^18^F-FDG uptake because of the tumor heterogeneity and some histopathology type (such as signet-ring cell carcinoma and mixed adenocarcinoma); and (3) some perigastric LNs were masked by the high ^18^F-FDG uptake of the primary tumor.

In the present study, the ^18^F-FDG PET/CT-based radiomics model showed a superior performance in discriminating LNMs with an AUC of 82.2% in the independent validation. Moreover, it also detected some LNMs that were missed in CECT (19.6%) and ^18^F-FDG PET/CT (35.7%), indicating that the PET/CT-based radiomics model could supplement ^18^F-FDG PET/CT to optimize the diagnostic performance. The performance might be attributed to the quantification process of the radiomic model applied for the final prediction and the parameters that could not be obtained by routine visual analysis and measurement of lymph node size and metabolism. Additionally, the correlation analysis indicated that the selected features (CT feature: Maximum3DDiameter; PET feature: Maximum2DDiameterSlice) used to establish the predictive model were significantly correlated to the pathological features, including vascular tumor thrombus, nerve invasion, and infiltration depth (p < 0.05). Since these pathological features were strongly associated with tumor invasion and metastasis, it could further explain the outstanding performance of the radiomic models.

Feng et al. proposed a clinical decision support system for the preoperative prediction of LNMs in GC (16) with the support vector machine (SVM) classifier. However, since the SVM classifier works by placing data points above and below the classifying hyperplanes, it would be difficult to generate a probabilistic explanation for the classification. Furthermore, the SVM would underperform in cases where the number of features for each data point exceeded the number of training data samples, which might be the reason for a large number of applied features (13) in the classifier. in the classifier. Our study employed an ensemble classifier to predict LNMs preoperatively. It improved the stability and the accuracy in the statistical classification and also helped reduce the variance to prevent overfitting. Thus, we achieved a better performance (accuracy 85.2% *vs*. 71.3%) with a smaller feature set (feature number 2 *vs*. 13) for the preoperative prediction of LNMs in GC.

Due to the low sensitivity and specificity, CECT and ^18^F-FDG PET/CT missed and incorrectly identified some LNMs. As a result, the performance of the two imaging modalities in predicting the number of LNMs was inferior. In comparison, the machine learning model showed a better predictive performance, with an overall AUC of 73.7% and an accuracy of 62.3% in the validation group. In addition, a proportion of patients in the validation group, whose LNMs were incorrectly staged by CECT (57.4%) and ^18^F-FDG PET/CT (55%), were then diagnosed correctly by our radiomic model, indicating that the radiomic model could supplement the current staging scheme. Dong et al. also reported a deep learning CT-radiomic model to predict the number of LNMs in GC with an overall C-index of 0.797 (0.771–0.823) (34). The model employed the deep learning features for delivering a high-quality result with the cost of the feature interpretability. Although with different methods, similar results indicated that the radiomic approach promised to facilitate an individualized prediction of N stages and help choose the best surgical approach with respect to resecting lymph nodes. Since the current study was a retrospective research, prospective research with GC patients recruited across multiple centers would be conducted in the future.

## 5 Conclusion

In this study in patients with GC, we successfully developed and validated machine learning models based on preoperative ^18^F-FDG PET/CT radiomics to identify LNMs and stratify patients into the different N stages. The machine learning model might be an important adjunct to conventional imaging modalities to help select the most appropriate treatment for patients with GC.

## Data Availability Statement

The original contributions presented in the study are included in the article/**Supplementary Material**, further inquiries can be directed to the corresponding authors.

## Ethics Statement

This study was approved by the Ethics Committee of the Fudan University Shanghai Cancer Center. The ethics committee waived the requirement of written informed consent for participation. Written informed consent was obtained from the individual(s) for the publication of any potentially identifiable images or data included in this article.

## Author Contributions

Methodology: QL, JL, and BX. Validation: JL, BX, and QL. Original draft preparation: QL and JL. Data collection: YS. Review and editing: MF. Visualization: all authors. Supervision and project administration: SS and XW. Funding acquisition: SS. All authors contributed to the article and approved the submitted version.

## Funding

This work was partially supported by the National Natural Science Foundation of China (Grant No. 82001866, 81971648, 81771861) and Shanghai Sailing Program (Grant No. 20YF1408400).

## Conflict of Interest

The authors declare that the research was conducted in the absence of any commercial or financial relationships that could be construed as a potential conflict of interest.

## Publisher’s Note

All claims expressed in this article are solely those of the authors and do not necessarily represent those of their affiliated organizations, or those of the publisher, the editors and the reviewers. Any product that may be evaluated in this article, or claim that may be made by its manufacturer, is not guaranteed or endorsed by the publisher.

## Abbreviations

AUC, area under the curve; CECT, contrast-enhanced computed tomography; CT, computed tomography; FDG, fluorodeoxyglucose; FN, false negative; FP, false positive; GC, gastric cancer; GLCM, gray-level co-occurrence matrix; GLSZM, gray-level size zone matrix; LIME, Local Interpretable Model-Agnostic Explanations model; LNMs, lymph node metastases; LoG, Laplacian of Gaussian; MTV, metabolic volume; N, nodal; NPV, negative predicted value; PET, positron emission tomography; PPV, positive predicted value; ROI, region of interest; sklearn, scikit-learn; SUV, standardized uptake value; SUVmax, the maximum standardized uptake value; TLG, total lesion glycolysis; VOI, volume of interest.
