# Supplementary figures and images for: 18F-FDG PET/CT Radiomics for Preoperative Prediction of Lymph Node Metastases and Nodal Staging in Gastric Cancer
Source: Front Oncol. 2021 Sep 13;11:723345. doi: 10.3389/fonc.2021.723345 (PMC8474469; doi:10.3389/fonc.2021.723345)

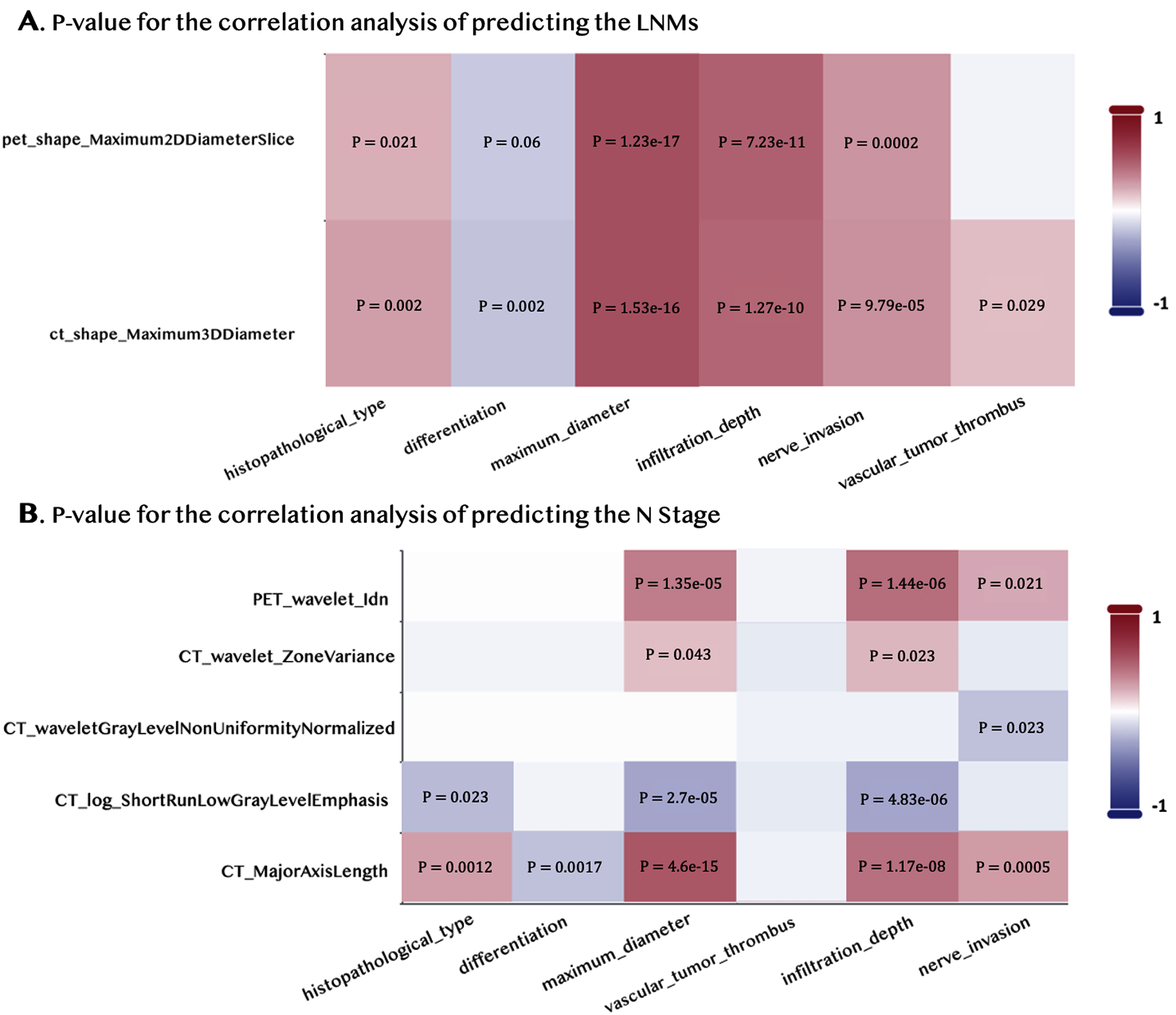

Supplement: Supplementary file 1 [file Image_1.jpeg]
