# Supplementary material for: 18F-FDG PET/CT Radiomics for Preoperative Prediction of Lymph Node Metastases and Nodal Staging in Gastric Cancer
Source: Front Oncol. 2021 Sep 13;11:723345. doi: 10.3389/fonc.2021.723345 (PMC8474469; doi:10.3389/fonc.2021.723345)
Supplement: Supplementary file 2 [file Table_1.docx]

**Table S1.** Statistics of the selected radiomic features for the prediction of LNMs.

| Radiomic Features | N0 | | | N1-3b | | P-value |  |
| --- | --- | --- | --- | --- | --- | --- | --- |
|  | mean ± std | range | mean ± std | | range |  | |
| ct_shape_Maximum3DDiameter | 57.5 ± 24.6 | 29.1 – 133.2 | 78.5 ± 28.9 | | 29.8 – 175.2 | 5.35e-05 | |
| pet_shape_Maximum2DDiameterSlice | 52.1 ± 21.9 | 20.8 – 115.6 | 68.5 ± 23.5 | | 20.8 –131.3 | 1.3e-04 | |
