# Supplementary material for: 18F-FDG PET/CT Radiomics for Preoperative Prediction of Lymph Node Metastases and Nodal Staging in Gastric Cancer
Source: Front Oncol. 2021 Sep 13;11:723345. doi: 10.3389/fonc.2021.723345 (PMC8474469; doi:10.3389/fonc.2021.723345)
Supplement: Supplementary file 3 [file Table_2.docx]

**Supplementary Table S2.** Definitions for the selected radiomic features for predicting the N stage.

| **Feature Name** | **Feature formula & definition** |
| --- | --- |
| ct_shape_MajorAxisLength | **Formula**: $major axis=4\sqrt{{}_{major}}$ , where ${}_{major}$ represents the largest principal component, which is performed using the physical coordinates of the pixel centers defining the ROI.  **Definition**: this feature measures the largest axis length of ROI enclosing ellipsoid. |
| ct_log-sigma-3-0-mm-3D_GLRLM_  ShortRunLowGrayLevelEmphasis | **Formula**: $SRLGLE=\frac{\sum_{i=1}^{N_{g}} \sum_{j=1}^{N_{r}} \frac{P\left( i,j \vert\theta\right)}{i^{2}j^{2}}}{N_{r}\left( \theta\right)}$ , where $N_{g}$is the number of discrete intensity values in the image, $N_{r}$ is the number of discrete run lengths in the image, $P\left( i,j \vert\theta\right)$ is the run length matrix for an arbitrary direction $\theta$, $N_{r}\left( \theta\right)$ is the number of runs in the image along angle $\theta$.    **Definition**: this feature measures the joint distribution of shorter run lengths with lower gray-level values. |
| ct_wavelet-HLH_GLRLM_  GrayLevelNonUniformityNormalized | **Formula**: $GLNN=\frac{\sum_{i=1}^{N_{g}} \left( \sum_{j=1}^{N_{r}} P\left( i,j \vert\theta\right) \right)^{2}}{N_{r}\left( \theta\right)^{2}}$ , where $N_{g}$ is the number of discrete intensity values in the image, $N_{r}$ is the number of discrete run lengths in the image, $P\left( i,j \vert\theta\right)$ is the run length matrix for an arbitrary direction $\theta$, $N_{r}\left( \theta\right)$ is the number of runs in the image along angle $\theta$.  **Definition**: this feature measures the similarity of gray-level intensity values in the image. |
| ct_wavelet-HHL_GLSZM_  ZoneVariance | **Formula**: $ZN=\sum_{i=1}^{N_{g}} \sum_{j=1}^{N_{s}} p\left( i,j \right)\left( j-\mu^{2} \right)$ , where $\mu=\sum_{i=1}^{N_{g}} \sum_{j=1}^{N_{s}} p\left( i,j \right)j$ , where $N_{g}$ represents the number of discrete intensity values in the image, $N_{s}$ is the number of discrete zone sizes in the image, $p\left( i,j \right)$ is the normalized zone size matrix.  **Definition**: this feature measures the variance in zone size volumes for the zones. |
| pet_wavelet-HHL_GLCM_Idn | **Formula**: $IDN=\sum_{k=0}^{N_{g}-1} \frac{p_{x-y}\left( k \right)}{1+\left( \frac{k}{N_{g}} \right)}$ , where $N_{g}$ is the number of discrete intensity levels in the image, $p_{x-y}(k) = \sum_{i=1}^{N_{g}} \sum_{j=1}^{N_{s}} p\left( i,j \right)$, $p\left( i,j \right)$ represents the normalized co-occurrence matrix.  **Definition**: Inverse difference normalized feature measures the local homogeneity of the image. |
